# *Humulus japonicus* attenuates LPS-and scopolamine-induced cognitive impairment in mice

**DOI:** 10.1186/s42826-022-00134-3

**Published:** 2022-07-19

**Authors:** Jun Go, Hye-Yeon Park, Da Woon Lee, So-Young Maeng, In-Bok Lee, Yun Jeong Seo, Jin-Pyo An, Won Keun Oh, Chul-Ho Lee, Kyoung-Shim Kim

**Affiliations:** 1 Laboratory Animal Resource Center, Korea Research Institute of Bioscience and Biotechnology, Gwahak-ro 125, Yuseong-gu, Daejeon, 34141 Republic of Korea; 2 Department of Biomaterials Science, College of Natural Resources and Life Science/Life and Industry Convergence Research Institute, Pusan National University, Miryang, 50463 Republic of Korea; 3 College of Biosciences and Biotechnology, Chung-Nam National University, Daejeon, 34134 Republic of Korea; 4 Korea Bioactive Natural Material Bank, Research Institute of Pharmaceutical Sciences College of Pharmacy, Seoul National University (SNU), Seoul, 08826 Republic of Korea; 5 Department of Functional Genomics, University of Science and Technology, Daejeon, 34113 Republic of Korea

**Keywords:** *Humulus japonicas*, Cognitive function, Neuroinflammation, Mouse model

## Abstract

**Background:**

Neuroinflammation plays an important role in cognitive decline and memory impairment in neurodegenerative disorders. Previously, we demonstrated that *Humulus japonicus* (HJ) has anti-inflammatory effects in rodent models of Alzheimer’s disease and Parkinson’s disease. The present study aimed to examine the protective potential of HJ extracts against lipopolysaccharide (LPS)-induced cognitive impairment and scopolamine-induced amnesia in mouse models. Cognitive improvement of mice was investigated by novel object recognition test. For analyzing effects on neuroinflammation, immunohistochemistry and quantitative real-time polymerase chain reaction (qRT-PCR) assays were performed.

**Results:**

We found that the oral administration of HJ significantly improved cognitive dysfunction induced by LPS in a novel object recognition test. The LPS-induced activation of microglia was notably decreased by HJ treatment in the cortex and hippocampus. HJ administration with LPS also significantly increased the mRNA expression of interleukin (IL)-10 and decreased the mRNA expression of IL-12 in the parietal cortex of mice. The increased expression of LPS-induced complement C1q B chain (*C1bq)* and triggering receptor expressed on myeloid cells 2 (*Trem2)* genes was significantly suppressed by HJ treatment. In addition, HJ administration significantly improved novel object recognition in a scopolamine-induced amnesia mouse model.

**Conclusions:**

These findings revealed that HJ has a beneficial effect on cognitive impairment and neuroinflammation induced by systemic inflammation and on amnesia induced by scopolamine in mice.

## Background

Systemic inflammation occurs when the immune system defends the body in a pro-inflammatory state [1]. Systemic inflammation can induce the generation of circulating cytokines, leading to immune responses in the brain [1]. Systemic inflammation is also known to affect cognitive functions [1–3]. Inflammatory processes contribute to the pathogenesis and progression of neurodegenerative disorders such as Alzheimer’s disease (AD) [4].

In experimental animal models using a direct peripheral challenge with the endotoxin lipopolysaccharide (LPS), systemic administration of the LPS has been shown to induce cognitive impairment in mice [5]. In clinical studies, administration of low doses of *Escherichia coli* LPS has been shown to negatively affect memory scores [2, 3]. LPS, which is released by bacteria, is a large molecule consisting of a polysaccharide and lipid [6] and can induce the activation of microglia and the production of pro-inflammatory cytokines in the brain [7]. The application of peripheral LPS also stimulates the activation of other molecular cascades in addition to pro-inflammatory cytokines, which may act as alternative mechanisms for memory impairment [8, 9]. Chemokines and amyloidogenic proteins have also been considered as alternative mediators [1, 5, 9]. In addition, other markers identified from transcriptional profiling of microglia have been used to define microglial phenotypes and neuroinflammation [10, 11].

*Humulus japonicus* (HJ) is a perennial herb known as ‘Japanese hop’ in Asian countries. Previous studies have reported that HJ provides protective effects against oxidative stress by scavenging active oxygen molecules, including hydrogen peroxide, hydroxyl radicals, and superoxide radicals [12, 13]. In vitro, HJ exerts anti-inflammatory effects by reducing inflammation-related molecules, including nitric oxide, inducible nitric oxide synthase and cyclooxygenase-2, and interleukin (IL)-6, through the regulation of nuclear factor of kappa light polypeptide gene enhancer in B-cells inhibitor, alpha phosphorylation in LPS-treated murine macrophage cell lines [14]. HJ treatment also reduced the LPS-induced mRNA expression of cytokines in murine microglial BV2 cells [15]. In an AD mouse model, HJ showed strong anti-inflammatory properties and attenuated pathophysiology, such as β-amyloid deposition, increased tau phosphorylation, and disease-related cognitive impairment of recognition memory and spatial working memory [15]. In the present study, we investigated whether HJ could attenuate cognitive impairment and alleviate microglial activation induced by systemic inflammation. Furthermore, scopolamine, a muscarinic cholinergic receptor antagonist,-induced amnesia is a widely used mouse model [16]. The cholinergic neurotransmission is involved in important physiological processes, such as learning process and attention [17, 18]. Degenerative changes of cholinergic neurons and cholinergic hypofunction during aging have been related to the memory deficit with aging [19]. In AD, cholinergic system has been suggested as an important factor in dementia and drugs targeting the cholinergic system have been used to treat AD with relative success [18, 20]. Scopolamine leads to cholinergic dysfunction and impaired cognition in rodents [21]. Mice with scopolamine-induced memory deficits have been used as animal models for screening potential anti-dementia agents [22]. Thus, the efficacy of HJ in cognitive function was confirmed in a scopolamine-induced amnesia mouse model.

## Results

### HJ improves novel object recognition in LPS-induced cognitive impairment

**Fig. 1:**
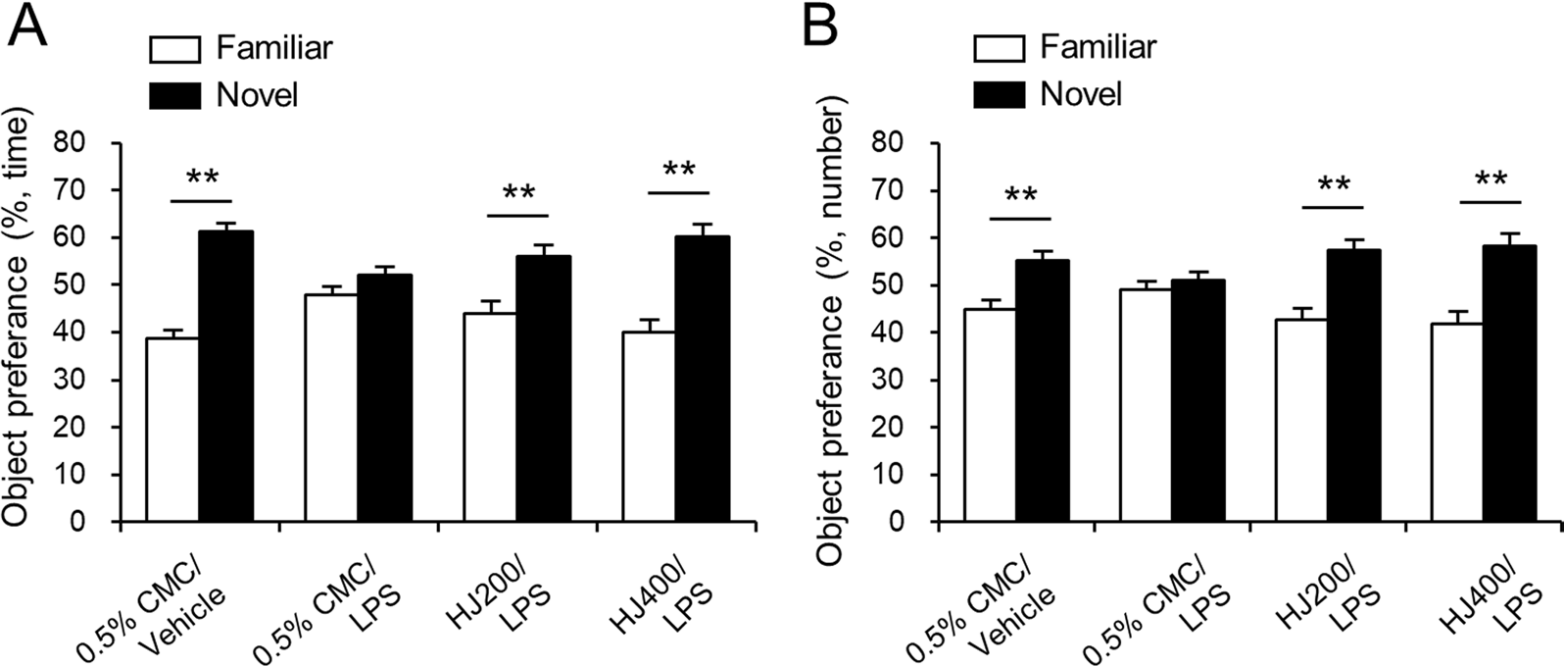
Effects of HJ on LPS-induced memory impairment in C57BL/6J mice. The preference for a novel object during the novel object recognition test was measured. The percentage of time spent sniffing (**A**) and number of touches (**B**) on the familiar and novel objects with the nose and/or forepaws is presented. **p < 0.01, significant differences between familiar and novel objects. Student’s t-test. 200 mg/kg HJ-treated mice (HJ200) and 400 mg/kg HJ-treated mice (HJ400). Data are presented as mean ± SEM

To investigate the memory-improving effects of HJ on LPS-induced cognitive impairment, mice received HJ treatment at a dose of 200 or 400 mg/kg/day daily for 7 days and then co-administered with 250 µg/kg/day LPS daily for 7 days. The oral administration of 200 or 400 mg/kg HJ successfully improved the novel object recognition memory in a mouse model of autism [23]. There was no difference in body weight among the 0.5% CMC/LPS-, HJ200/LPS-, and HJ400/LPS-treated groups on the last day of LPS injection (0.5% CMC/LPS, 22.2 g ± 0.3; HJ200/LPS, 22.2 g ± 0.3; HJ400/LPS, 22.8 g ± 0.2). A day after the last LPS injection, the NORT was performed. The total number of contacts and the total time spent sniffing with the novel object compared to the familiar object were measured. 0.5% CMC/vehicle-treated mice revealed more exploration preference for the novel object than for the familiar object, showing an increase in the time of sniffing and the number of touches on the novel object (Fig. 1A, B). 0.5% CMC/LPS-treated mice showed no significant difference in preference between familiar and novel objects. HJ treatment at 200 mg/kg or 400 mg/kg with LPS increased the exploration preference for the novel object (Fig. 1A, B). These results indicate that HJ administration induces protective effects against inflammation-induced cognitive impairment.

### HJ suppresses LPS-induced microglia activation in parietal cortex and hippocampus

**Fig. 2:**
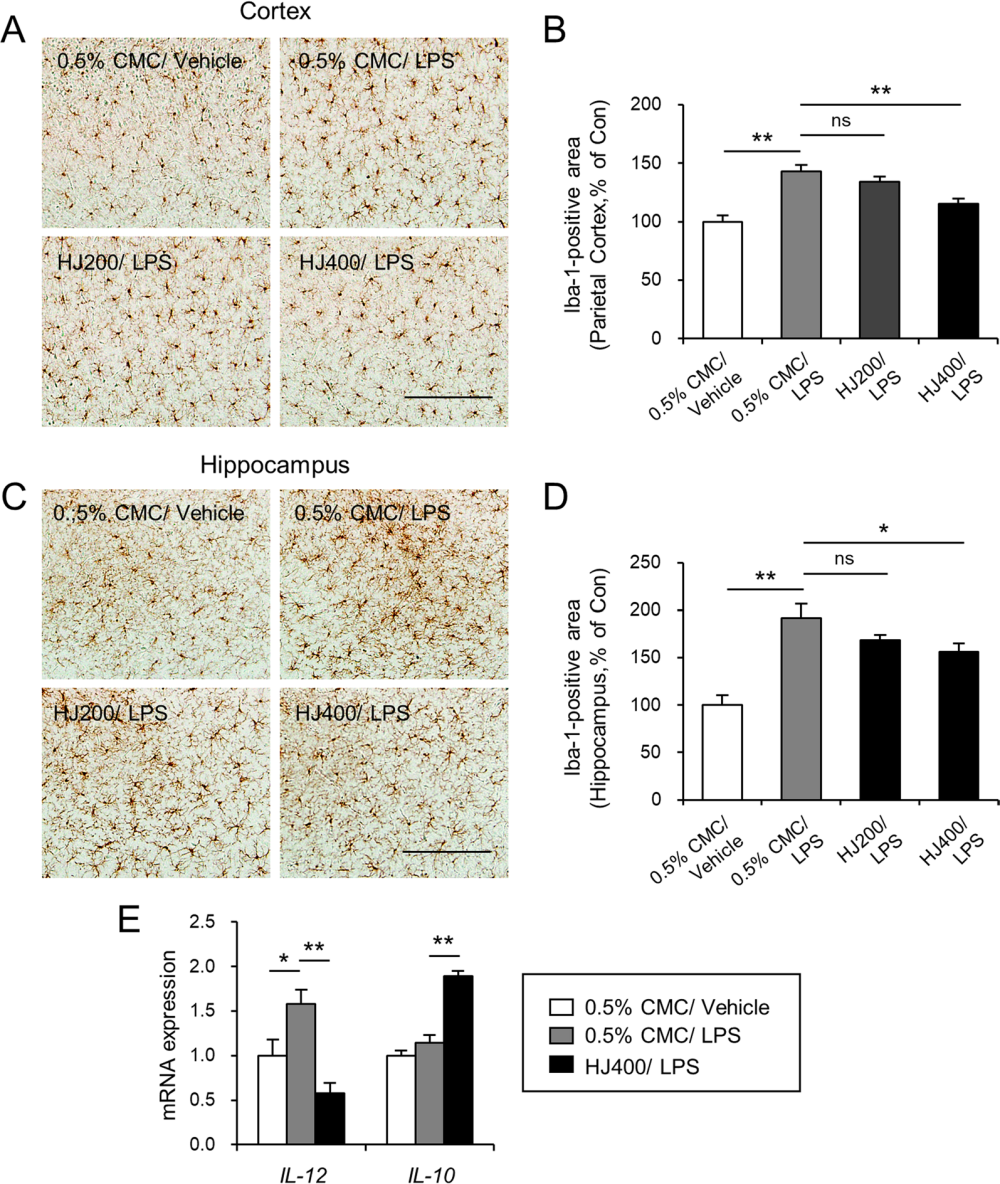
Effects of HJ on LPS-induced microglia activation in C57BL/6J mice. **A**, **C** Photomicrographs showing anti-Iba1 antibody-stained parietal cortex (**A**) and hippocampus (**C**). **B**, **D** Percent area occupied by Iba1 immunoreactivity in the parietal cortex (**B**) and hippocampus (D) in the vehicle + vehicle, vehicle + LPS, HJ200 (200 mg/kg HJ) + LPS, and HJ400 (400 mg/kg HJ) + LPS groups. **E** Fold change in mRNA expression of the *IL-12* and *IL-10* genes. *p < 0.05 and **p < 0.01, significant differences between indicated groups. ns, not significant. One-way ANOVA. Scale bar, 200 μm. Data are presented as the mean ± SEM

Previous studies have reported that intraperitoneal injection of LPS leads to activation of microglia in the parietal cortex and hippocampus [8, 24]. To investigate the effects of HJ on microglial activation, we measured the areas occupied by microglia in the parietal cortex and hippocampus using immunohistochemical assays (Fig. 2A, B). Systemic LPS administration markedly increased the number of activated microglia in the parietal cortex (Fig. 2A, *p* < 0.01) and hippocampus (Fig. 2C, *p* < 0.01). Although 200 mg/kg HJ treatment with LPS tended to decrease the percentage of occupied Iba-1 immunoreactive cells in both the parietal cortex and hippocampus (*p* > 0.05), 400 mg/kg HJ treatment with LPS significantly decreased the percentage of occupied Iba-1 immunoreactive cells in both the parietal cortex and hippocampus (Fig. 2B, *p* < 0.01; D, *p* < 0.05). To investigate whether the gene expression of *IL-10* (anti-inflammatory cytokine) and *IL-12* (pro-inflammatory cytokine) could be regulated by HJ treatment in the brain, real-time qPCR was performed in the parietal cortex of the mice. The mRNA expression of *IL-12* was increased by systemic LPS administration in mice, and the enhanced gene expression level was markedly suppressed by oral administration of HJ (Fig. 2E, *p* < 0.01). *IL-10* was not altered by LPS, but HJ treatment significantly increased its mRNA expression level in the parietal cortex (Fig. 2E, *p* < 0.01). These results demonstrate that HJ can suppress neuroinflammation in the parietal cortex and hippocampus.

### HJ suppresses LPS-induced mRNA expression of Trem2, C1qb, Cx3cr1, and Csf1 in the parietal cortex

**Fig. 3:**
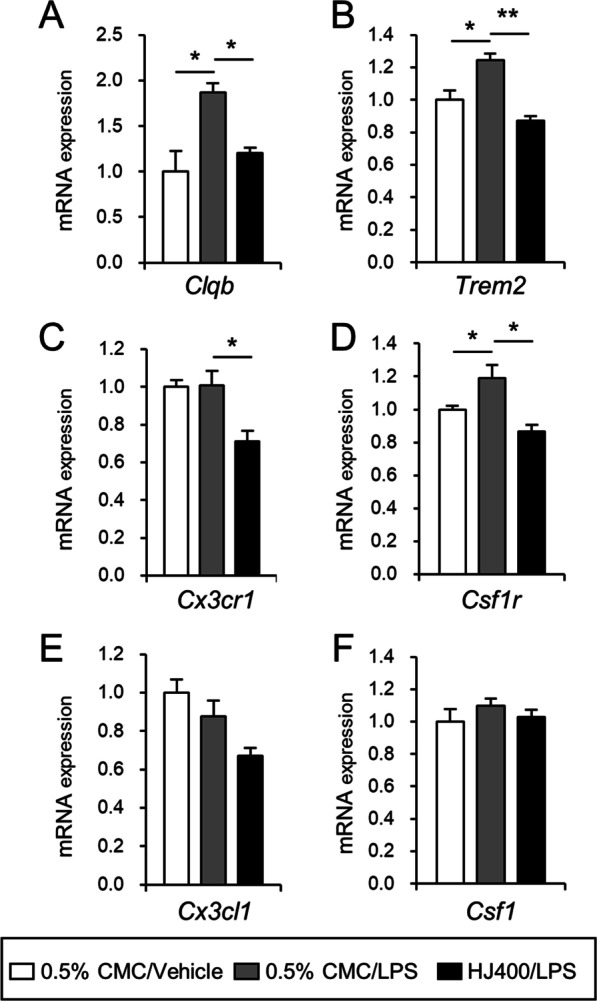
Effects of HJ on the mRNA expression related to changes in microglial phenotypes. **A**–**F** Fold changes in mRNA expression of *C1qb* (**A**), *Trem2* (**B**), *Cx3cr1* (**C**), *Csf1r* (**D**), *Cx3cl1* (**E**), and *Csf1* (**F**) genes. *p < 0.05 and **p < 0.01, significant differences between indicated groups. Student’s t-test and one-way ANOVA. Data are presented as the mean ± SEM

Recent studies have suggested that inflammatory changes in neurodegenerative disorders are related to changes in microglial phenotypes, such as homeostatic or disease-associated microglia [25, 26]. Neurodegeneration-related genes in microglia are associated with extracellular space proteins and the plasma membrane [11, 27]. To investigate the effect of HJ on the genes related to microglial phenotypes, mRNA expression of *Trem2, C1qb, Cx3cr1*, and *Csf1r* was analyzed in the parietal cortex of LPS-induced cognitive impairment mice. Interestingly, LPS administration significantly increased the mRNA expression of *Trem2*, *C1qb*, and *Csf1r* in the parietal cortex of mice compared with that in 0.5% CMC/vehicle-treated mice (Fig. 3A, B, and D). LPS-induced mRNA expression of *Trem2, C1qb, Cx3cr1*, and *Csf1r* was effectively suppressed by HJ treatment (Fig. 3A–D). The mRNA expression levels of *Cx3cl1* and *Csf1*, which are neuron-derived ligands of *Cx3cr1* and *Csf1r,* respectively, were not altered by LPS or HJ treatment (Fig. 3E, F).

### HJ improves novel object recognition in scopolamine-induced amnesia

**Fig. 4:**
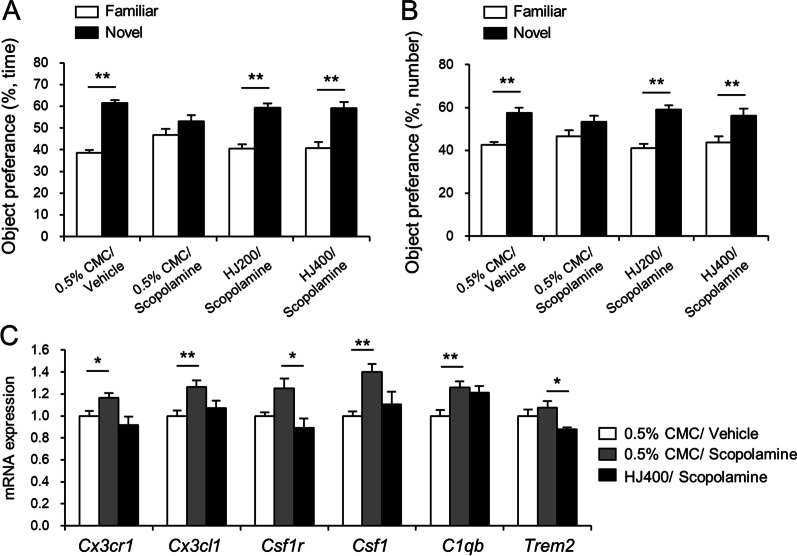
Effects of HJ on scopolamine-induced amnesia in C57BL/6J mice. The exploration preference for a novel object during the novel object recognition test was measured. The percentage of time spent sniffing (**A**) and number of touches **B** on the familiar and novel objects with the nose and/or forepaws is presented. **C** Fold changes in mRNA expression of *Cx3cl1, Cx3cr1, Csf1, Csf1r, C1qb*, and *Trem2* genes. *p < 0.05 and **p < 0.01, significant differences between indicated groups. 200 mg/kg HJ-treated mice (HJ200) and 400 mg/kg HJ-treated mice (HJ400). Student’s *t*-test. Data are presented as mean ± SEM

To confirm that the cognitive-improving effect of HJ on LPS-induced cognitive impairment was also revealed in another cognitive deficit model, scopolamine-induced amnesia mice were used. HJ or 0.5% CMC was administered 1 h before the behavioral experiment, and scopolamine or vehicle was administered 30 min before the NORT. Novel object recognition performance was measured in mice. The total number of contacts and the total time spent sniffing with the novel object compared to the familiar object were measured. 0.5% CMC/vehicle-treated mice revealed more exploration preference for the novel object than for the familiar object, showing an increase in the time of sniffing and the number of touches on the novel object (Fig. 4A, B). 0.5% CMC/scopolamine-treated mice showed no significant difference in preference between familiar and novel objects. Both 200 mg/kg and 400 mg/kg HJ treatments increased the exploration preference for the novel object (Fig. 4A, B). These results indicate that the administration of HJ showed protective effects against scopolamine-induced cognitive impairment.

To determine the effect of HJ on genes related to microglial phenotypes, mRNA expression of *Trem2, C1qb, Cx3cr1*, and *Csf1r* was analyzed in the parietal cortex of scopolamine-induced amnesia mice. Interestingly, 0.5% CMC/scopolamine administration significantly increased the mRNA expression of *C1qb, Cx3cr1*, and *Csf1r* in the parietal cortex of mice compared with that of 0.5% CMC/vehicle-treated mice (Fig. 4C). The mRNA expression levels of *Csf1r* and *Trem2* were effectively suppressed by HJ treatment (Fig. 4C). mRNA expression levels of *Cx3cl1* and *Csf1* also increased with scopolamine, and HJ administration induced a tendency to decrease the gene expression of both genes (Fig. 4C).

## Discussion

In the present study, LPS-induced systemic inflammation led to microglial activation in the brain as well as cognitive deficits in NORT. In addition, it was shown that the expression of genes related to changes in microglial phenotypes, such as homeostatic or disease-associated microglia, was induced by a direct peripheral challenge with LPS. Neuroinflammation is a nervous immune response specific to the central nervous system that causes glial cell activation in various neurodegenerative diseases, such as AD, Parkinson’s disease (PD), and amyotrophic lateral sclerosis [28, 29]. HJ treatment effectively improved the cognitive dysfunction induced by systemic LPS challenge or cholinergic receptor blocking.

Previously, we found that the mRNA expression and release of LPS-induced tumor necrosis factor-alpha (*TNF-α*)*, IL-1β*, and *IL-6* in the BV2 microglial cell line were significantly decreased by HJ treatment [15]. In addition, neuroinflammatory responses, such as microglial hyperactivation and increased expression levels of proinflammatory cytokines induced by β-amyloid deposits in an APP/PS1 transgenic mouse model of AD, were effectively suppressed by HJ administration [15]. Furthermore, HJ has a neuroprotective effect in a 6-hydroxydopamine (6-OHDA)-induced mouse model of PD [30]. Direct intracerebral injection of 6-OHDA leads to inflammatory responses and oxidative stress, and HJ administration exhibits strong antioxidant and anti-inflammatory effects [30]. HJ treatment also increased the anti-inflammatory cytokine IL-10 and decreased the pro-inflammatory cytokine *IL-12* levels in the cerebral cortex. Therefore, it is conceivable that increased *IL-10* and decreased *IL-12* expression may lead to anti-inflammatory effects.

C1q is involved in the upstream signaling of the complement pathway and regulates phagocytosis and pro-inflammatory signaling [31]. In the adult brain, C1q is normally downregulated, but it becomes upregulated in various diseases [32]. It has been suggested that unwanted synapses are tagged with complements for elimination in neurodegenerative diseases [32]. C1q suppresses pro-inflammatory cytokines, including TNF-α, IL-6, and IL-12, in LPS-stimulated bone marrow-derived macrophages and dendritic cells [33, 34]. An increase in C1QB mRNA has been reported in the cortex of brains with AD [35]. According to recent studies, *Trem2* plays a pivotal role in the differentiation of microglia into an activated state called damage-associated microglia in various neurodegenerative diseases such as AD and ALS [36–38]. In *Trem2* knockdown mice, microglia show impaired phagocytic activity and enhanced inflammatory responses [39]. Systemic LPS administration significantly upregulated *C1qb* and *Trem2* expression in the parietal cortex of mice, which was effectively suppressed by HJ treatment. Thus, it is possible that the suppressive effect of HJ on the expression of these genes has a protective effect in the brain.

Microglia communicate with neurons through direct cell-to-cell interactions [40]. It has been suggested that neurons can control microglial activation and motility, and microglia release signal molecules involved in the regulation of synaptic plasticity and neuronal activities [41]. Cell surface molecules and numerous receptors, such as CX3CR1 and CSF1R, of microglia can interact with cytokines secreted by neurons, such as the fractalkines CX3CL1 or CSF1 [42]. To investigate whether HJ treatment could alter neuron-microglia interactions, we assessed the expression levels of microglia-related genes, surface molecules, and neuron-secreted cytokines by qRT-PCR. LPS-induced systemic inflammation did not alter the expression of *Cx3cr1* and *Csf1r* in microglia. However, HJ administration with LPS significantly decreased the expression of these genes. In addition, *Csf1r* expression was decreased in the scopolamine-induced model. The mRNA expression of *Cx3cl1* or *Csf1* in neurons was not affected by LPS or HJ treatment in the parietal cortex. However, in the scopolamine-induced model, *Cx3cl1* and *Csf1* mRNA expression was significantly increased. CX3CL1-CX3CR1 interaction is involved in regulating synaptic plasticity, learning and memory [43] and social behaviors [44]. Moreover, Csf1r is expressed in macrophages, monocytes, and microglia, and is necessary for the development, maintenance, recruitment, and proliferation of microglia [45]. *Csf1r*-deficient mice showed a decrease in microglial density and function and died before adulthood [46–48]. Furthermore, the administration of selective CSF1R inhibitors in adult mice eliminated microglia [45]. These suggest the critical roles of CSF1R in microglial function. It is therefore necessary to determine whether the suppressive effect of HJ on *Cx3cr1* and *Csf1r* expression affects microglial function.

Recent study has shown that the ethanolic extracts of HJ contain luteolin-7-O-glucoside and apigenin-7-O-glucoside as the major compounds [49]. Luteolin and apigenin, natural flavonoids found in many plants, have been suggested as a therapeutic candidate for inflammation-related brain diseases [50, 51]. The compounds exhibited strong anti-inflammatory responses in interferon-gamma-treated primary microglia [52]. The compounds have been reported to have cognition-improvement effects [50, 53, 54]. In addition to the two flavonoids, Lee et al. isolated several purified and predicted compounds from the ethanolic extracts of HJ using HPLC-qTOF and NMR [49]. Further studies are needed to evaluate the effects of these compounds on cognitive impairment.

## Conclusions

Systemic inflammation and a chronic inflammatory state are largely considered to contribute to cognitive decline and cognitive dysfunction in the brain [1]. The findings of the present study demonstrate that HJ enhances novel object recognition during systemic LPS administration and has potential benefits in managing cognitive impairment. We also explored the underlying mechanisms, including the influence of microglial activation and alterations in the expression of inflammation-responsible genes. HJ treatment effectively suppresses neuroinflammation in the brain, and it is thought that the anti-inflammatory effect of HJ provides cognitive improvement. Further research is needed to determine how the suppressive effect of HJ on *Cx3cr1* and *Csf1r* gene expression affects neuron-microglia interaction. The cognitive improvement effect of HJ was also investigated in a scopolamine-induced amnesia mouse model. Because memory impairment induced by scopolamine is associated with increased oxidative stress as well as inflammation in the brain [21], related research on this topic is necessary.

## Methods

### Preparation of HJ

HJ was purchased from Gangwon Herbs (Gangwon, Korea). Prof. WK Oh identified the voucher specimen, and a specimen (SNU-2014-0004) was deposited at SNU in Korea. The ethanolic extract of HJ was prepared and supplied by the Korea Bioactive Natural Material Bank (Seoul, Korea). HJ preparation has been described previously [23]. The dried aerial parts of HJ were soaked in 20% ethanol in an extraction container for 2 days at room temperature (25 °C ± 2). The ethanol-soluble extract was filtered through cheesecloth, exhaustively concentrated, and dried to produce an ethanolic extract under reduced pressure. The extract of HJ was stored at room temperature (25 °C ± 2) until further use.

### Animals

Male C57BL/6J mice were purchased from Daehan Biolink (Chungbuk, Korea) and housed in regular polycarbonate plastic cages with controlled temperature (21–22 °C) and humidity (50–60%) as well as a 12-h light/dark cycle (lights on at 7 AM). Animals were maintained on an ad libitum diet of lab chow (Envigo Teklad, Madison, WI, USA). Mice had free access to water. The animal room was maintained under specific pathogen-free conditions. A ethanolic extract of HJ at 200 or 400 mg/kg/day or 0.5% carboxymethylcellulose (0.5% CMC, Sigma, St. Louis, MO, USA) was administered to mice by oral gavage once daily for 7 days, and then LPS (Sigma, St. Louis, MO, USA, 250 μg/kg/day) or saline (vehicle) was co-administered intraperitoneally (i.p.) 30 min after HJ or vehicle injection once a day for 7 days. Eight-week-old C57BL/6J mice were randomized into 0.5% CMC/vehicle (n = 11), 0.5% CMC/LPS (n = 10), HJ200/LPS (200 mg/kg of HJ/LPS, n = 8), and HJ400/LPS (400 mg/kg of HJ/LPS, n = 13) groups. A day after the last LPS injection, the novel object recognition test (NORT) was assessed for 2 days to evaluate learning and memory, and then 2 days later, mice were sacrificed for qRT-PCR and immunohistochemistry. In scopolamine-induced amnesia mouse model, HJ or 0.5% CMC was administered once 1 h before the behavioral experiment or qRT-PCR and scopolamine (Tocris, Bristol, UK, 1 mg/kg, i.p.) or saline (vehicle) was administered once 30 min before the behavior test or qRT-PCR. For the behavioral experiment, eight-week-old C57BL/6J mice were randomized into 0.5% CMC/vehicle (n = 12), 0.5% CMC/scopolamine (n = 12), HJ200/scopolamine (n = 14), and HJ400/scopolamine (n = 12) groups. For the qRT-PCR, eight-week-old C57BL/6J mice were divided into 0.5% CMC/vehicle (n = 8), 0.5% CMC/scopolamine (n = 8), and HJ400/scopolamine (n = 8) groups. The parietal cortex was immediately dissected 30 min after scopolamine injection and stored in a − 80 °C deep freezer until qRT-PCR analysis. All mice experiments were approved by the Institutional Animal Use and Care Committee of the KRIBB (KRIBB-AEC-18004).

### Quantitative real-time polymerase chain reaction analyses (qRT-PCR)

In this study, parietal cortex, known to be involved in attention as well as spatial learning and memory [55–57], was used for qRT-PCR. RNA preparation and qRT-PCR were performed as previously described [58]. Briefly, the parietal cortex was used to evaluate the expression levels of complement c1q B chain (*C1qb*), CX3C chemokine receptor 1 (*Cx3cr1*), triggering receptor expressed on myeloid cells 2 (*Trem2*), colony stimulating factor 1 (*Csf1*), CSF1 receptor (*Csf1r*), IL-12, IL-10 and CX3C chemokine ligand 1 (*Cx3cl1*) by qRT-PCR. The difference in the amplification fold was calculated based on qRT-PCR amplification of the target gene against 18 s ribosomal RNA as a reference. Total RNA was purified from the parietal cortex tissue using the TRI reagent (Sigma-Aldrich, St. Louis, MO, USA). Reverse transcription was performed using the Promega RT-PCR kit (Promega, Madison, WI, USA). The PCR reaction was prepared with 5 μl of 2 × SYBR Green mix (Applied Biosystems, Poster City, CA, USA) and performed using the StepOne Real time PCR system (Applied Biosystems). The cycling conditions were as follows: 10 min at 95 °C followed by 40 cycles of 20 s at 95 °C, 30 s at 60 °C, and 20 s at 72 °C. The following primer sets were used: *IL-10* (5′-GGG TTG CCA AGC CTT ATC G-3′ and 5′-TCT CAC CCA GGG AAT TCA AAT G-3′), *IL-12* (5′-TGG TTT GCC ATC GTT TTG CTG -3′ and 5′-ACA GGT GAG GTT CAC TGT TTC T-3′), *Cx3cl1* (5′-CGC GTT CTT CCA TTT GTG TA-3′ and 5′-TGG GAT TCG TGA GGT CAT CT-3′), *Cx3cr1* (5′-GAG TAT GAC GAT TCT GCT GAG G-3′ and 5′-CAG ACC GAA CGT GAA GAC GAG-3′), *Csf1* (5′-GGC TTG GCT TGG GAT TCT-3′ and 5′-GAG GGT CTG GCA GGT ACT C-3′), *Csf1r* (5′-TGT CAT CGA GCC TAG TGG C-3′ and 5′-CGG GAG ATT CAG GGT CCA AG-3′), *C1qb* (5′-TCT GGG AAT CCA CTG TC-3′ and 5′-AGA CCT CAC CCC ACT GTG TC-3′), *Trem2* (5′-CTG GAA CCG TCA CCA TCA CTC-3′ and 5′-CGA AAC TCG ATG ACT CCT CGG-3′), and *18 s* (5′-GAC ACG GAC AGG ATT GAC AGA TTG ATA G-3′ and 5′-GTT AGC ATG CCA GAG TCT CGT TCG TT-3′).

### Novel object recognition test (NORT)

NORT is widely used to evaluate recognition memory [59–62]. Mice were individually placed in a testing chamber (40 × 20 × 20 cm^3^) for 10 min with two identical objects (familiar). A day later, the mice were placed back in the testing chamber with one of the familiar objects and one novel object for 10 min. Cylindrical wooden blocks were the identical familiar objects, whereas a rectangular wooden block was the novel object. All sessions were video recorded, and an observer who was blinded to the drug treatment measured the number of times the objects and the time spent exploring the objects were touched. The objects and chambers were cleaned with ethanol between the trials.

### Immunohistochemistry

Immunohistochemistry was performed as previously described [58]. The brains were dissected and fixed in 4% paraformaldehyde. The fixed brain was cut into 40 μm coronal sections on a vibratome (VT1000S, Leica, Wetzlar, Germany). The sections were incubated in tris-buffered saline (TBS) containing 3% H_2_O_2_ and rinsed 3 times with TBS-0.1% Tween 20. The sections were blocked with serum for 1 h at room temperature (25 °C ± 2) and incubated overnight at 4 °C with anti-ionized calcium-binding adapter molecule 1 (Iba-1, Wako, Osaka, Japan). The sections were incubated in an avidin/biotin ABC complex (ABC kit, Vector Laboratories, Burlingame, CA, USA) with biotinylated anti-rabbit IgG secondary antibody (Vector Laboratories). All sections were treated with 3,3′-diaminobenzidine (Sigma-Aldrich), mounted on microscope slides, and analyzed with a light microscope (Olympus Corporation, Tokyo, Japan). Iba-1-stained areas in the parietal cortex and hippocampus were assessed using MetaMorph (Molecular Devices Inc., Sunnyvale, CA, USA).

### Statistical analysis

Statistical analyses were performed using GraphPad Prism (GraphPad Software, Inc., San Diego, CA, USA). Multiple comparisons were made using one-way ANOVA followed by Tukey–Kramer’s post hoc test. Two-sample comparisons were carried out using two-tailed Student’s *t*-test. All data were presented as the mean ± standard error of the mean (SEM). Statistical differences were accepted at the 5% level unless otherwise indicated.

## Data Availability

All data generated or analyzed during this study are included in this article. Further enquiries can be directed to the corresponding author.

## Abbreviations

6-OHDA: 6-Hydroxydopamine
AD: Alzheimer’s disease
*C1qb*: Complement C1q B chain
*Csf1*: Colony stimulating factor 1
*Csf1r*: CSF1 receptor
*Cx3cl1*: CX3C chemokine ligand 1
*Cx3cr1*: CX3C chemokine receptor 1
HJ: *Humulus japonicus*
Iba-1: Ionized calcium-binding adapter molecule 1
IFN- γ: Interferon-gamma
*IL*: Interleukin
LPS: Lipopolysaccharide
NORT: Novel object recognition test
PD: Parkinson’s disease
qRT-PCR: Quantitative real-time polymerase chain reaction
TBS: Tris-buffered saline
TBS-T: TBS-0.1% Tween 20
*TNF-α*: Tumor necrosis factor-alpha
*Trem2*: Triggering receptor expressed on myeloid cells 2
